# Associations Between Gut Microbiota and Fecal Semiochemical and Metabolic Profiles in Sows During the Periparturient Period

**DOI:** 10.3390/ani16091346

**Published:** 2026-04-28

**Authors:** Yuansheng Wu, Haoran Yan, Chuchen Gui, Zhaokun Chen, Xiangdong Liu, Lingna Zhang

**Affiliations:** 1 Laboratory of Companion Animal Science, Department of Animal Science, South China Agricultural University, Guangzhou 510642, China; chocolatycone@163.com (Y.W.); 13680730396@163.com (H.Y.); m18978024616@163.com (C.G.); 13640744747@163.com (Z.C.); 2 Guangxi Yangxiang Group Co., Ltd., Guigang 537131, China

**Keywords:** sow, periparturient period, gut microbiota, fecal semiochemicals, metabolic profiles, coprophagy

## Abstract

The attraction of neonatal piglets to sow feces and their coprophagy suggests that fecal semiochemicals promote nutrient intake and gut colonization, but their microbial origin is poorly understood. This study explored associations between gut microbiota and fecal semiochemical/metabolic profiles in prepartum and postpartum sows. Fecal samples were collected from six crossbred sows at 4 days prepartum and 3 days postpartum. Nutritional components, volatile organic compounds (VOCs), untargeted metabolomics, and gut microbiota were analyzed. Nutritional composition remained largely stable postpartum, except that sodium increased while potassium, magnesium, and cellulose decreased. Skatole and p-cresol concentrations increased postpartum even after dry matter correction. Untargeted metabolomics identified 298 differentially abundant metabolites (250 up, 48 down) postpartum, enriched in nicotinamide/nicotinate metabolism, arginine biosynthesis, and others. Gut microbial alpha diversity (Shannon, Chao1, observed features) decreased postpartum. Fibrolytic and butyrate-producing bacteria (e.g., *Ruminococcaceae UCG-005*, *Lachnospira*) were enriched prepartum, whereas *Escherichia-Shigella*, *Christensenellaceae R-7* group and *Enterococcus* were more abundant postpartum. Spearman correlations showed skatole and p-cresol were strongly negatively correlated with butyrate-producing bacteria and positively correlated with postpartum-enriched genera. This study uncovered prepartum-to-postpartum shifts in sow fecal components and microbiota, revealing a microbial basis for semiochemical production and offering probiotic insights using coprophagy to improve piglet health.

## Simple Summary

Newborn piglets are naturally attracted to their mother’s feces and will eat small amounts—a behavior known as coprophagy. Scientists believe this helps the piglets obtain important nutrients and probiotics to start building a healthy gut. However, it is unclear what exactly in the sow’s feces has triggered this behavior. In this study, we compared feces from sows collected prepartum and postpartum. We found that while most basic nutrients stayed the same, the levels of specific chemical compounds increased significantly after postpartum. These smelly compounds are likely the signals that attract the piglets. The study also revealed that this change was linked to a shift in the types of bacteria living in the sow’s gut; postpartum, there was an increase in bacteria like *Escherichia-Shigella* and a decrease in bacteria that produce beneficial substances. These findings suggest that the mother’s gut microbes control the production of these attractive scents. By managing the sow’s gut health, we might be able to enhance these natural signals, encouraging piglets to eat more of the feces and ultimately improve their early growth and long-term health.

## 1. Introduction

Coprophagy, the behavior of eating feces, has been reported in mammals such as rodents, horses, swine and some non-human primates [1,2,3,4]. Aviles-Rosa et al. addressed the importance of this behavior in piglets, showing that deprived access to maternal feces compromised their immunocompetence and growth performance [5]. Coprophagy may provide benefits, especially in newborns, by supplying key nutrients, minerals and energy, and facilitating microbial transfer, which may aid in the establishment of a functional gut microbiome [1,6]. In the natural environment, nursing piglets have been estimated to consume an average of 20 g/d of maternal feces [3]. However, in modern intensive production systems, piglets typically have limited access to sow feces due to biosecurity concerns and hygiene management practices.

Newborn piglets are naturally attracted to maternal feces, and the fecal matter of postpartum sows demonstrates greater attractiveness to piglets compared to that of prepartum sows, implying the presence of potential semiochemicals in postpartum sow feces that mediate this behavior [5,7,8]. Skatole and myristic acid have been proposed as potential key components of maternal fecal semiochemicals, which is supported by the observed increase in their concentration in sow feces postpartum and their attractiveness to piglets in preference assessment [8,9,10]. Furthermore, experimental application of these compounds to feeders has been shown to reduce aggression with the tendency to promote feeding behavior in weaned pigs, indicating potential roles in stress management [8,9,10].

Studies have investigated the temporal and spatial variability in microbiomes in sows and growing pigs and their correlations with host phenotypes and different treatments [11,12,13,14]. These findings address and provide insight into the importance of optimizing stage and context-specific probiotics. So far, the connections between temporal changes of gut microbiome and fecal metabolites and the volatiles from prepartum to postpartum in sow remain unexplored. Considering the existence of potential maternal semiochemicals in sow feces and the exhibition of coprophagy in newly born piglets, this preliminary study aimed to evaluate the associations between gut microbiota and fecal semiochemical profiles and metabolites in sows before and after parturition. We therefore hypothesized that postpartum-enriched bacteria produce fecal semiochemicals that attract piglets to exhibit coprophagy, and this study aimed to test this hypothesis by correlating microbial shifts with volatile metabolite changes. It was thought that the findings might result in a better understanding of the mechanism under the functions of maternal semiochemicals and offer a new perspective towards the development of probiotics for newborn piglets.

## 2. Materials and Methods

### 2.1. Animals

All experimental procedures were approved and conducted in accordance with the Guidelines for Care and Use of Laboratory Animals of South China Agricultural University (2025G003). Fecal samples were collected from six pregnant sows (Yorkshire × Landrace, 201.68 ± 1.30 kg; parity 2–4) at a commercial pig farm (affiliated with Guangzhou Pucheng Biological Technology Co., Ltd., Guangzhou, China) in Jiangmen City, Guangdong Province, China. Sows were fed in compliance with the National Research Council’s nutritional guidelines. During prepartum, sows were fed a corn–soybean meal gestation diet formulated to provide 2.25 Mcal NE/kg and 15.0% crude protein. During late prepartum and postpartum, sows were fed a lactation diet formulated to provide 2.40 Mcal NE/kg, 17.0% crude protein, and 1.1% lysine. They were housed individually in conventional stalls (2.2 m × 0.6 m × 1.0 m) from breeding day 85, where they were provided restricted feeding (3.0–3.5 kg/d) and fed twice daily. Starting from day 100 of prepartum (approximately two weeks before the expected farrowing day), sows were switched to lactation feed and fed the same amount as during the prepartum period, twice daily at 07:00 and 16:00. Sows were moved to the farrowing crates (2.2 m × 1.5 m × 1.0 m) on day 108 of prepartum. The dietary ingredients and nutrient contents of the gestation and lactation feed are shown in Supplementary Material (Supplementary Tables S1–S4). After parturition, the feed allowance was gradually increased by 1.0 kg per day until the sows were fed ad libitum. Feed was provided as a water-soaked mash, and water was available ad libitum via nipple drinkers. The temperature of the farrowing house was maintained at 25 ± 2 °C in summer, with warming lamps installed locally to maintain the temperature for piglets.

### 2.2. Fecal Collection

Fecal samples were repeatedly obtained from the same sows. Prepartum group samples (N = 6, *n* = 18) were collected for three consecutive days beginning four days before farrowing. Postpartum group (N = 6, *n* = 18) samples were collected for three consecutive days starting three days after farrowing. All samples were obtained directly from the rectum at a similar time in the morning prior to feeding. Immediately after collection, feces were frozen in liquid nitrogen and transported to the laboratory. Upon arrival, aliquots of 2 g were prepared for the analysis of volatile compounds and nutritional profiling. Aliquots were stored at −80 °C, and volatile analysis was conducted within two weeks to minimize loss of volatiles.

### 2.3. Volatile Compound Extraction and Analysis

Volatile compounds were extracted from fecal samples using solid-phase microextraction (SPME) with a 50/30 μm DVB/CAR/PDMS StableFlex fiber (SUPELCO) [15]. One gram of homogenized sample was placed in a 20 mL headspace vial and sealed. The fiber was conditioned at 250 °C for 300 s prior to extraction. Samples were pre-incubated at 40 °C, followed by extraction at 60 °C for 2400 s with agitation at 300 rpm (600 s active stirring). The fiber was exposed at 15 mm insertion depth with 12 mm coating extension, then desorbed at 270 °C for 300 s in the GC inlet. Analysis was performed on an Agilent 7890A gas chromatograph (Agilent Technologies, Santa Clara, CA, USA) coupled to a 5975C mass spectrometer. Separation was achieved using an HP-5MS capillary column (30 m × 0.25 mm × 0.25 μm, Agilent Technologies) with helium carrier gas at 1 mL/min. The oven temperature program was as follows: 40 °C (hold 5 min), ramped at 5 °C/min to 70 °C (hold 2 min), then 5 °C/min to 150 °C, followed by 25 °C/min to 230 °C (hold 4 min). The injector was maintained at 270 °C in splitless mode with a 3 mL/min septum purge. Mass spectrometry detection employed electron impact ionization (70 eV) with the ion source at 230 °C, quadrupole at 150 °C, and transfer line at 280 °C. Data were acquired in scan mode (*m*/*z* 30–450) [16].

### 2.4. Nutritional Analyses

Samples collected on different days in each period (i.e., prepartum and postpartum) were composited as one sample of individual sow for nutritional analysis. Fecal samples were processed according to Chinese National Standards to determine the proximate components including dry matter (DM, GB/T 6435-2014) [17], crude protein (CP, GB/T 19438-2008) [18], crude fiber (CF, GB/T 6434-2022) [19], and ash (GB/T 6438-2007) [20]. The neutral detergent fiber (NDF) and acid detergent fiber (ADF) were determined using Van Soest sequential extraction with an ANKOM 2000i automated fiber analyzer [21]. The measured micronutrient contents included calcium (Ca, GB/T 6436-2018) [22], total phosphorus (P, GB/T 6437-2018) [23], and sodium (Na) and potassium (K, GB/T 13885-2017) [24].

### 2.5. Untargeted Metabolomics

Metabolite extraction followed the method of previous studies [25]. Specifically, an accurately weighed aliquot of each sample was placed in a 2 mL centrifuge tube, followed by the addition of 600 µL of methanol containing 2-chloro-L-phenylalanine (4 ppm, stored at −20 °C). The mixture was vortexed for 30 s, and 100 mg of glass beads was added before homogenization in a tissue grinder at 60 Hz for 90 s. After ultrasonication at room temperature for 10 min, the samples were centrifuged at 12,000 rpm and 4 °C for 10 min. The supernatant was filtered through a 0.22 μm membrane, and the filtrate was transferred to an injection vial for liquid chromatography–mass spectrometry (LC–MS) analysis.

Chromatographic separation was performed on a Thermo Ultimate 3000 UHPLC system (Thermo Fisher Scientific, Waltham, MA, USA) equipped with an ACQUITY UPLC^®^ HSS T3 column (2.1 × 150 mm, 1.8 µm; Waters, Milford, MA, USA) at a flow rate of 0.25 mL/min and a column temperature of 40 °C. The injection volume was 2 µL. For positive ion mode, the mobile phases consisted of (C) 0.1% formic acid in acetonitrile and (D) 0.1% formic acid in water, with the following gradient program: 0–1 min, 2% C; 1–9 min, 2–50% C; 9–12 min, 50–98% C; 12–13.5 min, 98% C; 13.5–14 min, 98–2% C; 14–20 min, 2% C. For negative ion mode, the mobile phases were (A) acetonitrile and (B) 5 mM ammonium formate in water, with the following gradient program: 0–1 min, 2% A; 1–9 min, 2–50% A; 9–12 min, 50–98% A; 12–13.5 min, 98% A; 13.5–14 min, 98–2% A; 14–17 min, 2% A.

MS detection was performed using a Thermo Q Exactive mass spectrometer (Thermo Fisher Scientific, USA) with an electrospray ionization (ESI) source operating in both positive and negative ion modes. The spray voltages were set at 3.50 kV (positive) and −2.50 kV (negative), with sheath gas at 30 arb and auxiliary gas at 10 arb. The capillary temperature was maintained at 325 °C. Full-scan MS spectra were acquired at a resolution of 70,000 over a mass range of *m*/*z* 100–1000. Data-dependent MS/MS acquisition was performed using higher-energy collisional dissociation (HCD) with a normalized collision energy of 30% and a resolution of 17,500. The top 10 most intense ions were selected for fragmentation, with dynamic exclusion enabled to minimize redundant MS/MS acquisition.

### 2.6. Analysis of Microbiota

Total genomic DNA was extracted from samples using the magnetic bead-based soil and fecal genomic DNA extraction kit (TianGen, Beijing, China) and quantified via 1% agarose gel electrophoresis. Target regions (16S rRNA V4) were amplified with barcoded primers 515F (5′-GTGCCAGCMGCCGCGGTAA-3′) and 806R (5′-GGACTACHVGGGTWTCTAAT-3′). PCR reactions were performed in 15 μL reactions containing Phusion High-Fidelity PCR Master Mix (New England Biolabs, Ipswich, MA, USA), 2 μM each primer and ~10 ng DNA. Cycling conditions were as follows: 98 °C for 1 min; 30 cycles of 98 °C/10 s, 50 °C/30 s, 72 °C/30 s; final 72 °C/5 min.

PCR products were verified by 2% agarose gel electrophoresis, purified using magnetic beads and quantified by enzyme-labeled assay. Equimolar amounts of purified products were pooled, and the pooled library was constructed using the NEB Next Ultra II FS DNA PCR-free Library Prep Kit (New England Biolabs). The library was quantified with Qubit 2.0 and Agilent Bioanalyzer 2100 and sequenced on an Illumina NovaSeq 6000 platform with 2 × 250 bp paired-end reads.

Raw reads were demultiplexed, merged with FLASH (v1.2.7) [26], quality-filtered per QIIME (v1.9.1), and chimeras removed via VSEARCH v2.15.0 against SILVA v138.1 databases [27]. Sequences were clustered into operational taxonomic units (OTUs) at 97% similarity using UPARSE (v7.0.1001). Taxonomy was assigned using SILVA with Mothur v1.48.0/RDP classifier v2.13 [28]. Multiple sequence alignment was performed with MUSCLE (v3.8.31) [29]. OTU tables were rarefied to the lowest depth. Alpha diversity (Observed species, Chao1, ACE, Shannon, Simpson, Good’s coverage) was calculated in QIIME (v1.7.0) and visualized in R (v2.15.3).

### 2.7. Data Analysis

All data were analyzed using IBM SPSS Statistics 26.0 (IBM Corp., Armonk, NY, USA). Normality and homogeneity of variance were assessed using Shapiro–Wilk and Levene’s tests, respectively. Paired-sample *t*-tests or Wilcoxon signed-rank tests were used to compare prepartum and postpartum differences in nutritional components, α-diversity indices, and volatile organic compounds (VOCs) concentrations (both wet-basis and DM-corrected). To specifically evaluate the effect of fecal moisture variation on key semiochemicals, an analysis of covariance (ANCOVA) was performed using fecal dry matter percentage as a covariate when comparing skatole and p-cresol concentrations between the two periods. Differential metabolites, microbial diversity, LEfSe, and Spearman correlations were analyzed as described previously. The analytical procedure consisted of three main steps. Kruskal–Wallis rank-sum test was applied to detect all potential taxa with significant abundance differences between groups. Wilcoxon rank-sum test was used to assess whether all subspecies of the identified taxa belonged to the same taxonomic rank. Linear discriminant analysis (LDA) was conducted to determine the final biomarkers (differentially abundant taxa). All tests were two-tailed, with significance declared at *p* < 0.05 and high significance at *p* < 0.01. Results are presented as mean ± standard error (SE). Spearman’s rank correlation analysis was used to evaluate associations between gut microbiota and fecal volatile compounds. Pairwise Spearman’s correlation coefficients (ρ) were calculated between the relative abundances of significantly altered microbial taxa and all the identified 32 fecal volatile compounds, using the “corr.test” function in the “psych” package or the base “corr.test” function (method = “spearman”). *p*-values were adjusted for multiple comparisons using the Benjamini–Hochberg false discovery rate (FDR) method. Correlations with |ρ| > 0.6 and FDR-adjusted *p* (q-value) < 0.05 were considered significant and visualized as a heatmap using the “pheatmap” or “ComplexHeatmap” package, with hierarchical clustering of rows (microbial taxa) and columns (odor-related compounds) [30].

## 3. Results

### 3.1. Fecal Nutritional Value

Although postpartum sows exhibited reduced feed intake, the nutrient composition of their feces remained largely unchanged. Notably, Na concentration increased, while K and Mg concentration and cellulose content significantly decreased (Table 1).

### 3.2. Fecal Semiochemicals

A total of 32 VOCs were identified during prepartum and postpartum, with no differences found in the types of fecal volatiles and no unique volatiles identified for the two phases. When comparing the peak area ratio of volatiles, 13 compounds exhibited significant differences in fecal content between prepartum and postpartum (*p* < 0.05). Specifically, seven volatiles increased (*p* < 0.05) postpartum, including skatole, p-cresol, 1-butanol (3-methyl), tetradecane, pentadecene, toluene, and cyclopentasiloxane (decamethyl-), while six volatiles significantly decreased postpartum, including carbamic acid (monoammonium salt), cyclohexasiloxane (dodecamethyl-), 2-methylbutanoic acid, dimethyl disulfide, and cycloheptasiloxane (tetradecamethyl-). Standard curves of target chemicals were used to estimate the concentrations of the top five VOCs that had increased in the postpartum sow feces (Table 2). Among these, only skatole and p-cresol exhibited significant increase during postpartum compared to prepartum, when calculated based on wet feces and after correcting for differences in fecal dry matter.

### 3.3. Fecal Metabolites

**Figure 1:**
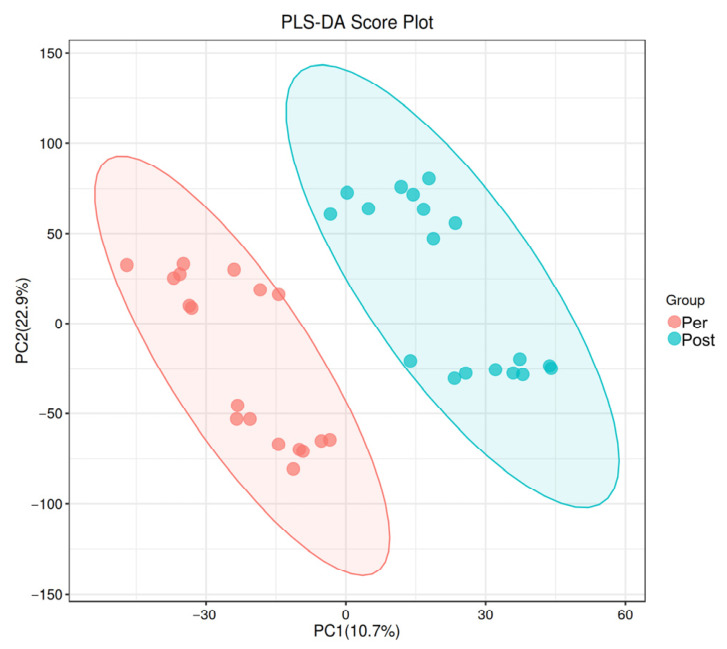
PLS-DA Score Plot showing separation of samples between groups. PC1 (*x*-axis) and PC2 (*y*-axis) represent the first and second principal components, respectively. Dots indicate individual samples, colored by group; ellipses denote 95% confidence regions. Pre: Prepartum group (*n* = 18). Post: Postpartum group (*n* = 18).

**Figure 2:**
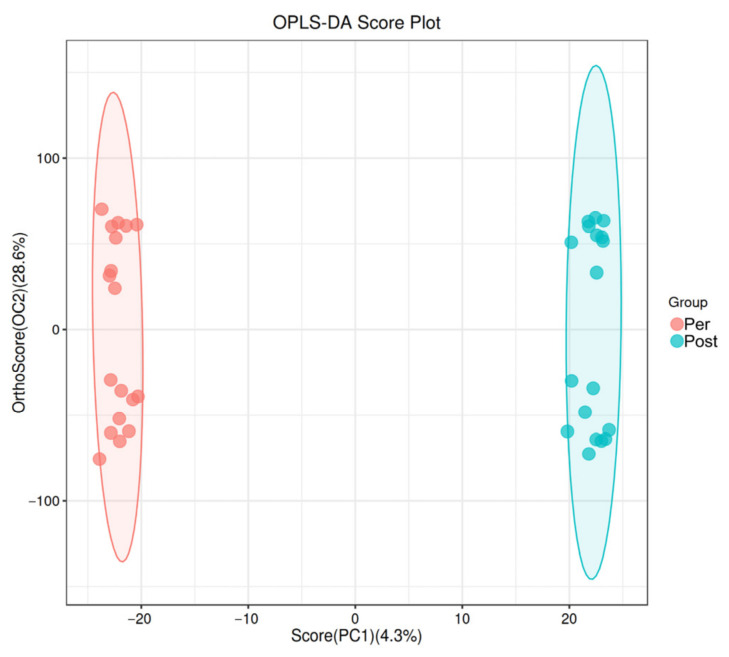
Orthogonal partial least squares discriminant analysis (OPLS-DA) score plot of fecal samples from prepartum and postpartum sows. The *x*-axis (PC1) represents the score of the first predictive component, reflecting between-group differences, while the *y*-axis (OC2) represents the score of the first orthogonal component, reflecting within-group differences. Each point corresponds to an individual experimental sample, with colors indicating different groups (e.g., red: postpartum sows; blue: prepartum sows). Pre: Prepartum group (*n* = 18). Post: Postpartum group (*n* = 18).

**Figure 3:**
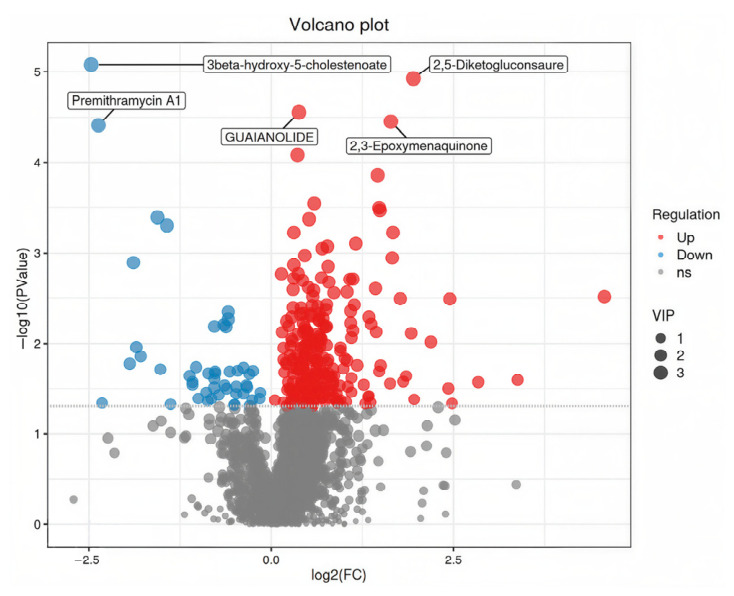
Volcano plot of differentially expressed fecal metabolites between prepartum and postpartum sows (prepartum group, *n* = 18; postpartum group, *n* = 18). The *x*-axis represents the log_2_-transformed fold change (log_2_FC) of metabolite abundance between the two groups, and the *y*-axis represents the negative log_10_-transformed *p*-value (−log_10_(*p*-value)). Each point corresponds to an individual metabolite. Greater absolute values on the *x*-axis indicate larger fold differences in expression levels between groups, while higher values on the *y*-axis indicate greater statistical significance and higher reliability of the differentially expressed metabolites. Point size reflects the variable importance in projection (VIP) value from OPLS-DA. Red points denote significantly upregulated metabolites, blue points denote significantly downregulated metabolites, and gray points represent metabolites that did not meet the differential screening criteria (e.g., |log_2_FC| ≥ 1 and *p* < 0.05). The top 5 metabolites with the lowest *p*-values are labeled with their *m*/*z* values by default.

**Figure 4:**
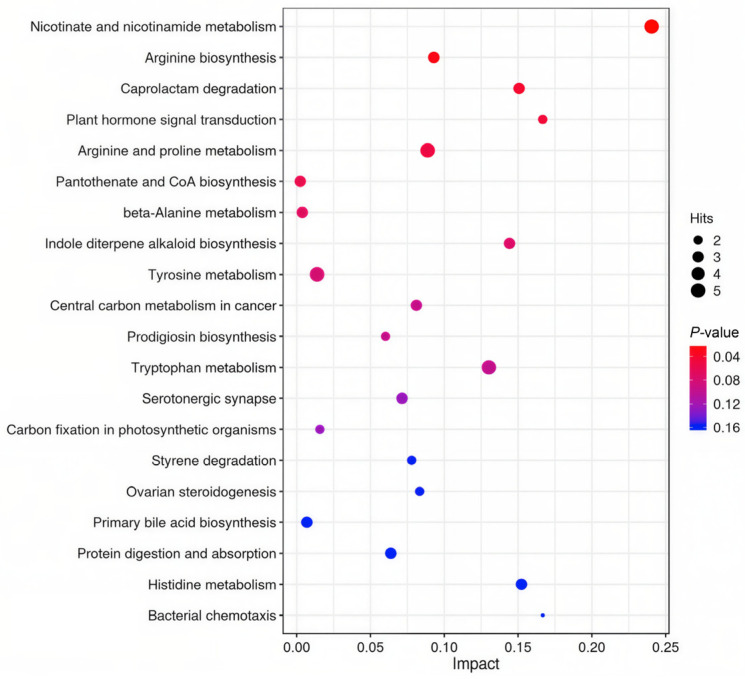
Bubble plot of enriched metabolic pathways based on differentially expressed fecal metabolites (prepartum group, *n* = 18; postpartum group, *n* = 18). The *x*-axis represents the pathway impact value (derived from topological analysis), and the *y*-axis shows the enriched metabolic pathways. Bubble size indicates the number of matched metabolites mapped to each pathway. Bubble color reflects the enrichment significance based on *p*-value, with red indicating smaller *p*-values (higher significance) and blue indicating larger *p*-values (lower significance).

The PLS-DA and OPLS-DA score plots revealed significant differences in fecal metabolites between prepartum and postpartum sows (Figure 1 and Figure 2). Furthermore, a total of 298 differentially abundant metabolites were identified at the secondary level, of which 250 were upregulated and 48 were downregulated (Figure 3). The Kyoto Encyclopedia of Genes and Genomes (KEGG) enrichment analysis demonstrated that these differential metabolites were primarily enriched in pathways related to nicotinamide and nicotinate metabolism, arginine biosynthesis, caprolactam degradation, plant hormone signal transduction, as well as arginine and proline metabolism (Figure 4).

### 3.4. Fecal Microbiota

**Figure 5:**
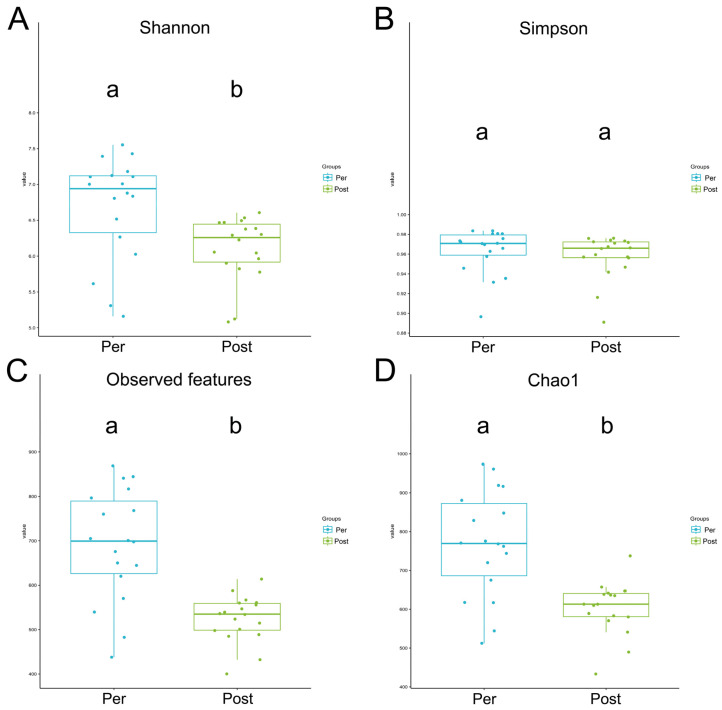
Alpha diversity analysis of gut microbiota in prepartum and postpartum sows. Boxplots showing four alpha diversity indices: (**A**) Shannon index, (**B**) Simpson index, (**C**) Observed features, and (**D**) Chao1 index. Each index was calculated at the genus level (or ASV level) based on rarefied sequencing data. Boxes represent the interquartile range (IQR), with the line inside indicating the median; whiskers extend to 1.5 × IQR. Individual data points are overlaid as dots. Statistical significance between groups was determined by the Kruskal–Wallis rank-sum test (Values with the same letter are not significantly different, while those with different letters are significantly different). Pre: Prepartum group (*n* = 18). Post: Postpartum group (*n* = 18). The same letters (e.g., ‘aa’) indicate no significant difference, while different letters (e.g., ‘ab’) indicate a significant difference.

The Shannon index decreased in sow microbiota postpartum (*p* < 0.05), while the Simpson index showed no significant difference (Figure 5A,B). Additionally, the Chao1 index and observed-feature index also decreased significantly postpartum (Figure 5C,D). These results indicate that both microbial diversity and total microbial abundance in the gut microbiota were significantly altered between the prepartum and postpartum stages.

**Figure 6:**
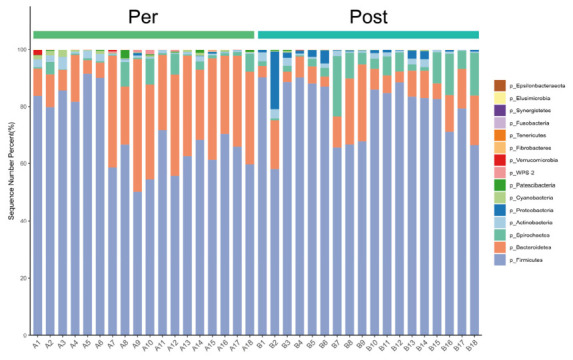
Stacked bar plot showing the relative abundance of bacterial phyla in fecal/gut samples from prepartum and postpartum groups. Each bar represents an individual sample, with samples grouped by condition (prepartum on the left, postpartum on the right). Colors correspond to different phyla as indicated in the legend (major phyla including Bacteroidetes, Firmicutes, Proteobacteria, and others shown individually; low-abundance phyla may be included in minor segments). Pre: Prepartum group (*n* = 18). Post: Postpartum group (*n* = 18).

The results showed that both groups were predominantly composed of *Firmicutes*, *Bacteroidetes*, *Spirochaetes*, *Actinobacteria*, and *Proteobacteria* (Figure 6). LEfSe analysis was performed to identify differences in taxa abundance between prepartum and postpartum stages in sows. At the genus level, the taxa of *Ruminococcaceae UCG-005*, *Lachnospira*, *Anaerovibrio*, *[Eubacterium] hallii group*, *Marvinbryantia*, *Subdoligranulum*, *Moryella*, *Acetitomaculum*, *Coprococcus 1*, and *[Eubacterium] xylanophilum group* exhibited higher relative abundance prepartum compared to postpartum. In contrast, the genera that were more abundant postpartum were *Ruminococcaceae UCG-002*, *Christensenellaceae R-7 group*, *Escherichia-Shigella*, *Family XIII AD3011 group*, *Ruminococcaceae UCG-009*, *Enterococcus*, *Clostridiales bacterium* enrichment *culture clone 06-1235251-67*, *Weissella*, *UBA1819*, and Olsenella* (Figure S1).

### 3.5. Correlations Between Fecal Metabolites and Microbiota

**Figure 7:**
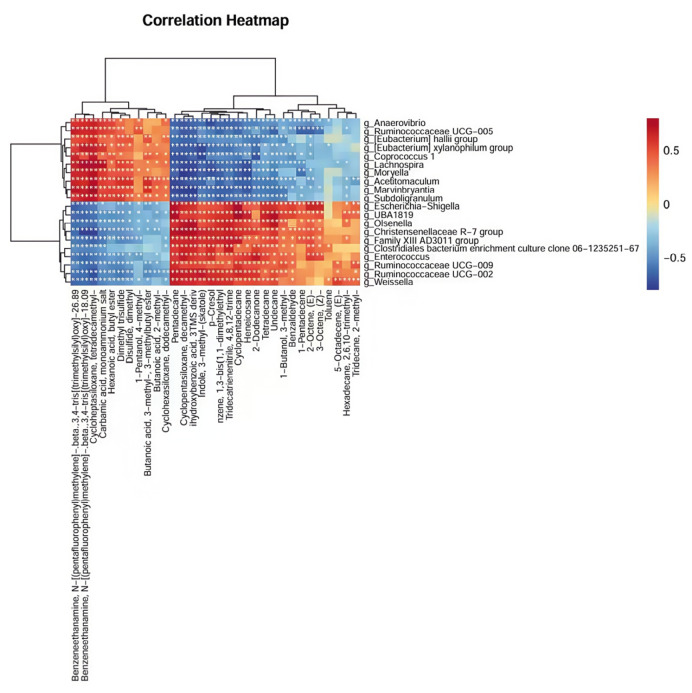
Spearman correlation heatmap illustrating the relationships between fecal volatile compounds and bacterial genera (prepartum group, *n* = 18; postpartum group, *n* = 18). The color gradient represents Spearman’s correlation coefficients (ρ), ranging from −1 (blue, strong negative correlation) to +1 (red, strong positive correlation). Significant correlations are marked with asterisks (* *p* < 0.05, ** *p* < 0.01). Compounds are shown on the *y*-axis and bacterial genera on the *x*-axis.

The Spearman correlation heatmap illustrates the correlations between the volatile compounds and differential microbial taxa in the sow feces (Figure 7). Skatole and p-cresol show strong negative correlations with butyrate-producing bacteria, including *Ruminococcaceae UCG-005*, *Lachnospira*, *[Eubacterium] hallii group*, and *Subdoligranulum* (r < −0.65, *p* < 0.001), while showing strong positive correlations with *Escherichia-Shigella*, *Ruminococcaceae UCG-002*, *Christensenellaceae R-7 group*, *Enterococcus*, *Weissella*, and others (r > 0.60, *p* < 0.001).

## 4. Discussion

This study analyzed fecal samples from prepartum sows to reveal the transformation process of the nutrient elements from late prepartum to early postpartum. Despite reduced feed intake postpartum, the nutrient composition in feces remained largely stable, with only the sodium level increased, while the potassium ion and cellulose content significantly decreased. The switch to lactation feed occurred two weeks before farrowing, providing sufficient time for dietary adaptation prior to sampling, which indicates that dietary shifts are unlikely to be the primary cause of nutritional, microbial, and metabolic changes. The observed changes in fecal mineral content appear to stem from the specific nutritional and physiological regulatory mechanisms during late prepartum [31]. The patterns of fecal sodium and potassium concentrations demonstrate how the body adjusts mineral metabolism in late prepartum to support maternal electrolyte balance and mineral supply in milk (e.g., potassium) for the postpartum period [32]. The switch of diet might still have influenced the fecal cellulose content. The hormonal fluctuations during the prepartum period may also play a key role, with elevated prolactin levels postpartum and changes in hormones such as estrogen and progesterone leading to alterations in intestinal permeability, mucus production, and immune responses, thereby modulating the gut microbiota and related metabolites [13,33].

The PCoA plot showed clear separation between fecal microbiota communities during late prepartum and early postpartum, characterized by reduced alpha diversity, such as decreases in the Shannon and Chao indices over time. Specifically, microbiota in the late prepartum period was enriched at the genus level with traditional fiber-degrading and butyrate-producing bacteria, including *Ruminococcaceae UCG-005*, *Lachnospira*, *[Eubacterium] xylanophilum group*, *Subdoligranulum*, *Anaerovibrio*, *Coprococcus 1*, and *[Eubacterium] hallii group*. These genera specialize in breaking down complex plant polysaccharides to produce short-chain fatty acids (e.g., butyrate and propionate), which provide over 70% of the energy for intestinal epithelial cells and regulate inflammation and systemic metabolic processes for maintaining the integrity of the intestinal barrier [34,35]. Meanwhile, genera such as *Christensenellaceae R-7 group*, *Weissella*, *Escherichia-Shigella*, *Ruminococcaceae UCG-002*, *Enterococcus*, *Family XIII AD3011 group*, *Weissella* and *UBA1819* substantially increased in the first week postpartum compared to late prepartum. The gut microbiota undergoes non-random shifts due to the synergistic effects of various physiological factors around farrowing [33]. The decline in progesterone level along with other elevated hormone levels (e.g., prolactin, growth hormone, IGF-1, and cortisol) in the early postpartum period resulted in the accelerated apoptosis, proliferation and shedding of intestinal epithelial cells and mucosal renewal [9,36,37]. Fecal cellulose content in postpartum also declined after farrowing, potentially due to the dietary shifts. The reduced intestinal motility and prolonged fecal retention time were caused by elevated progesterone levels inhibiting smooth muscle contraction during late prepartum; this creates an environment conducive to the proliferation of proteolytic bacteria and opportunistic pathogens. These factors collectively lead to a rapid shift in hindgut fermentation patterns from glycolytic–butyrate type to proteolytic–putrefactive type, which is supported by previous studies [14,36,38]. KEGG pathway enrichment analysis of differential metabolites revealed significant enrichment of postpartum-upregulated metabolites in pathways, including nicotinic acid and nicotinamide metabolism, arginine biosynthesis, arginine and proline metabolism, caprolactam degradation, and phytohormone signaling. Downregulated metabolites were fewer in number and did not form significantly enriched pathways. Upregulation of nicotinic acid and nicotinamide metabolism may enhance NAD+/NADH synthesis, thereby supporting cellular energy production and antioxidant defense to meet metabolic demands during postpartum and to alleviate oxidative stress [39].

Enrichment in arginine biosynthesis and arginine/proline metabolism provides precursors for nitrate and polyamine synthesis, promoting intestinal epithelial cell proliferation, tissue repair, and vascular health [40]. In alignment with these rapid changes, postpartum hormonal shifts occurred, including elevated prolactin, growth hormone, cortisol, and IGF-1, resulting in increased demand for amino acid derivative metabolites and accelerated intestinal mucosal renewal [40]. Activation of the caprolactam degradation pathway also indicates enhanced proteolytic fermentation [41,42]. Enrichment in phytohormone signaling pathways may stem from microbial metabolism of plant-derived compounds in the feed or represent an indirect response to altered gut microenvironments [43]. The changes of metabolic profile in sow feces from late prepartum to early postpartum provide supportive evidence to the shifts of fecal microbiota communities. These changes in the gut microbiota composition were largely consistent with the observed shifts in metabolic pathways, particularly the upregulation of nicotinic acid and nicotinamide metabolism, arginine biosynthesis, and arginine and proline metabolism, which collectively support the increased metabolic demands and physiological adaptations during the postpartum period.

The types of the identified VOCs in sow feces did not alter between prepartum and postpartum periods, while the significant changes of abundance occurred with 13 compounds. The significant elevation in skatole and p-cresol concentrations in the postpartum period, even after dry matter correction signifies the potentially critical roles in postpartum sows and newborn piglets. Another study partially verified our results which identified skatole and myristic acid as the key semiochemicals in the sow feces after farrowing [8]. Factors such as the varied sampling timepoints, dietary content, and analytical protocols might have contributed to the difference in the results of the two studies. Nevertheless, skatole was the VOC agreed upon, and p-cresol is one of the downstream products of skatole metabolism [44]. A computational study that assessed the binding affinity and complex stability between compounds of sow feces and porcine olfactory receptor proteins indicated that skatole and p-cresol can function as maternal semiochemicals at natural concentrations [45]. By forming stable hydrogen bonds and hydrophobic interactions with odorant-binding proteins (OBP) and salivary lipocalins (SAL), these compounds can be transported to the main olfactory system and activate downstream neuroendocrine pathways, leading to reduced cortisol levels and improved behavioral performance in piglets, thereby helping to alleviate weaning stress [45]. When skatole and p-cresol bind to aryl hydrocarbon (AhR) and pregnane X receptors, the activation of these receptors leads to increased expression of CYP1A1 and UGT1A1 enzymes, thereby protecting the liver from oxidative stress during high lipid mobilization in postpartum sows [46]. Skatole and p-cresol also regulate intestinal hormones GLP-1 and PYY, resulting in improved intestinal motility during postpartum and reduced constipation in the sow [47]. At low concentrations, p-cresol acts to inhibit the NF-κB signaling pathway and to decrease COX-2 mRNA expression, leading to reduced systemic inflammation [48]. The dynamic nature of sow fecal chemistry around farrowing is likely driven by alterations in microbial activity. As shown by the Spearman correlation heatmap, we identified positive correlations between skatole and p-cresol and genera such as *Escherichia-Shigella*, *Ruminococcaceae UCG-002*, *Christensenellaceae R-7 group*, *Enterococcus*, and *Weissella*. The finding agrees with a previous study, where Skatole production was found to primarily originate from microbial tryptophan metabolism, mainly involving Firmicutes and Bacteroidetes phyla [49]. Studies with preference assessment have proposed that semiochemicals of postpartum sow feces may promote coprophagy in newborn piglets [5,7]. In addition, the application of key volatiles to the feeders increased feeding and reduced aggression in weaning pigs [5]. Coprophagy may promote microbiota colonization and immune development in piglets, as studies have shown that deprivation of sow feces is associated with impaired autonomous growth and immune competence [5]. Neonatal piglets might ingest fresh sow feces to obtain essential nutrients, microbes, as well as maternal-level downstream products of skatole, including indole-3-acetic acid (IAA), indole-3-propionic acid (IPA), as well as p-cresol [50]. As mentioned earlier, these compounds may serve as natural AhR ligands that trigger the production of antioxidant genes HO-1 and NQO1 in intestinal epithelial cells, leading to enhanced expression of tight junction proteins, Treg cell development, mucosal barrier maturation, and immune tolerance establishment in piglets within 48 h post-birth [51].

This research provides insights for developing targeted probiotics for sows and newborn piglets, utilizing genera positively associated with semiochemical production to enhance the benefits of natural coprophagy. Genera of *Ruminococcaceae UCG-002*, *Christensenellaceae R-7 group*, *Enterococcus*, *Weissella*, which showed strong positive correlations with skatole and p-cresol in the analysis, have been suggested to exhibit probiotic potentials in swine [52,53,54,55]. Multi-strain probiotics incorporating these microbes can be administered to piglets to enhance semiochemical attractiveness, promote maternal microbiota transfer, and reduce reliance on antibiotics amid escalating resistance concerns. Future investigations should prioritize strain-specific evaluations in randomized controlled trials, optimizing dosages, timing (e.g., immediate postpartum or weaning application), and interactions with periparturient stressors to maximize efficacy in commercial settings.

Due to biosecurity and animal welfare regulations on commercial farms, this study could not conduct in vivo experiments to investigate the effects of the recommended probiotic strains (e.g., *Enterococcus* and *Weissella*, positively correlated with skatole and p-cresol) and their semiochemicals on piglet coprophagy, gut microbiota colonization, immune development, or growth performance. This limitation prevented us from inferring causal relationships from the observed correlations. Additional limitations include the following: (1) although sows were all parity 2–4 to minimize age-related variation, parity-dependent microbiota differences may still exist, and the small sample size did not allow us to include parity as a covariate, so the prepartum-to-postpartum microbial shifts could be partially confounded by parity or age; (2) this is a preliminary study without an independent validation cohort, so findings should be interpreted as hypothesis-generating; (3) the study used a single management system and diet, limiting generalizability. Future research should validate these microbial–metabolite associations in larger, multi-site, and parity-diverse populations, and test whether manipulating these microbes (e.g., with probiotics) can enhance piglet health through natural coprophagy. Moreover, once ethical approvals and farm collaborations are secured, it would be desirable to conduct controlled feeding trials or oral probiotic interventions in sows or piglets to further explore causality and to help develop antibiotic-independent strategies for piglet health management.

## 5. Conclusions

This study demonstrated that the prepartum to postpartum period in sows is associated with significant shifts in both gut microbial composition and fecal chemical profiles. The increased concentrations of skatole and p-cresol in postpartum feces, alongside the enrichment of specific bacterial genera such as *Escherichia-Shigella*, suggest a microbial mechanism underlying the production of volatile semiochemicals that may attract neonatal piglets. The concurrent decrease in fibrolytic and butyrate-producing bacteria highlights a trade-off in microbial function during this physiological transition. These findings provide preliminary evidence that maternal gut microbiota regulates the production of fecal compounds potentially involved in piglet coprophagy. From a practical perspective, this work opens new avenues for developing targeted probiotics that could enhance beneficial microbial functions in prepartum to postpartum sows, thereby supporting neonatal gut health and growth through natural behavior. Further research is warranted to establish causal relationships and explore translational applications.

## Figures and Tables

**Table 1:** Nutrient composition of sow feces across the prepartum and postpartum period.

Nutrient (Based on Fresh Feces)	Prepartum (*n* = 18)	Postpartum (*n* = 18)	SE ^a^	*p*-Value ^b^
Moisture, %	62.64	64.18	1.48/1.03	0.332
DM, %	37.36	35.82	1.48/1.03	0.332
CP, %	14.06	13.62	0.38/0.34	0.443
Fat, %	1.81	1.83	0.13/0.12	0.937
Ash, %	24.07	25.53	1.15/1.30	0.398
Hemicellulose, %	10.92	11.28	0.44/0.42	0.565
Cellulose, %	15.97	14.24	0.62/0.24	0.017
Lignin, %	21.36	22.10	0.35/0.26	0.113
ADF, %	37.33	36.34	0.60/0.39	0.196
NDF, %	48.25	47.62	0.74/0.59	0.552
Ca, %	0.96	0.82	0.07/0.05	0.109
P, %	0.73	0.60	0.05/0.04	0.057
Mg, %	0.29	0.22	0.02/0.02	0.022
K, %	0.33	0.25	0.02/0.01	0.005
Na, %	0.019	0.042	0.003/0.005	0.004
Cu, ppm	32.8	26.3	3.5/2.7	0.158
Zn, ppm	212	170	20/17	0.123
Fe, ppm	309	230	43/36	0.192

^a^ SE standard error of the difference. ^b^ Significance level of the effect of period (prepartum and postpartum). Abbreviations: DM, dry matter; CP, crude protein; ADF, acid detergent fiber; NDF, neutral detergent fiber; SE, standard error; ppm, parts per million; Ca, calcium; P, phosphorus; Mg, magnesium; K, potassium; Na, sodium; Cu, copper; Zn, zinc; Fe, iron.

**Table 2:** Concentrations of volatiles increased in lactating sow feces based on peak area of LC–MS analysis.

Volatiles	Basis (μg/g)	Prepartum (*n* = 18) (Mean ± SD)	Postpartum (*n* = 18) (Mean ± SD)	*p*-Value
p-Cresol	Wet	162.5 ± 79.3	398.7 ± 89.4	0.0015
	DM	465.2 ± 192.6	1371.8 ± 341.7	0.0021
Skatole	Wet	1.6 ± 2.7	35.1 ± 13.9	<0.0001
	DM	4.2 ± 5.4	127.4 ± 41.2	<0.0001
Tetradecane	Wet	9.7 ± 8.4	18.4 ± 10.9	0.19
	DM	28.1 ± 21.3	55.9 ± 23.8	0.24
Toluene	Wet	4.5 ± 7.0	13.2 ± 15.7	0.21
	DM	13.2 ± 13.1	49.8 ± 56.4	0.13
Decamethylcyclopentasiloxane	Wet	2.4 ± 1.5	11.8 ± 9.1	0.082
	DM	6.4 ± 3.8	33.7 ± 23.7	0.11

## Data Availability

The 16S rRNA data were deposited in the NC-BI repository, accession number: https://www.ncbi.nlm.nih.gov/, accessed on 8 March 2026, PRJNA1433047.

## Supplementary Materials

The following supporting information can be downloaded at https://www.mdpi.com/article/10.3390/ani16091346/s1; Table S1. Lactation Feed Formula; Table S2. Nutrient Content of Lactation Feed; Table S3. Gestation Feed Formula; Table S4. Nutrient Content of Gestation Feed. Figure S1. LDA effect size bar plot of differentially abundant microbial taxa between prepartum and postpartum groups.

## Abbreviations

The following abbreviations are used in this manuscript:

ADF	Acid detergent fiber
CF	Crude fiber
CP	Crude protein
DM	Dry matter
FDR	False discovery rate
KEGG	Kyoto Encyclopedia of Genes and Genomes
LDA	Linear discriminant analysis
LC-MS	Liquid chromatography–mass spectrometry
LEfSe	Linear discriminant analysis effect size
NDF	Neutral detergent fiber
OTU	Operational taxonomic unit
PCoA	Principal coordinates analysis
SE	Standard error
VOC	Volatile organic compound
