# Supplementary material for: Associations Between Gut Microbiota and Fecal Semiochemical and Metabolic Profiles in Sows During the Periparturient Period
Source: Animals (Basel). 2026 Apr 28;16(9):1346. doi: 10.3390/ani16091346 (PMC13163083; doi:10.3390/ani16091346)
Supplement: Supplementary file 1 [file animals-16-01346-s001.zip › animals-4238406-supplementary.pdf]

Table S1. Lactation Feed Formula

Ingredient	Percentage (%)
Corn	60.93
Wheat bran	8.0
Soybean hulls	3.3
43% Soybean meal	15.7
Salt	0.4
Limestone	1.2
Dicalcium phosphate	1.08
Soybean oil	2.0
60% Choline chloride	0.1
Baking soda	0.25
L-Lysine	0.11
DL-Methionine	0.1
L-Threonine	0.04
L-Valine	0.03
Glucose	0.5
Fish meal	1.0
Premix (micro minerals + vitamins)	0.2
Phytase	0.01
Fermented soybean meal	—
**Total**	**100.0**

Table S2. Nutrient Content of Lactation Feed

Nutrient	Level
Crude protein	17.0%
Crude fat	4.92%
Crude ash	5.95%
Crude fiber	4.04%
Calcium	1.0–1.2% (≈1%)
Total phosphorus	0.62%
Sodium	0.26%
Digestible phosphorus	0.38%
Digestible lysine	0.90%
Digestible methionine + cystine	0.65–0.68%
Digestible threonine	0.55%
Digestible tryptophan	0.21%
Digestible valine	—
**Net energy**	**2409 kcal/kg**

Table S3. Gestation Feed Formula

Ingredient	Percentage (%)
Corn	54.16
Wheat bran	18.0
Soybean hulls	7.4

43% Soybean meal	16.0
Salt	0.4
Limestone	0.93
Dicalcium phosphate	1.0
Soybean oil	1.5
60% Choline chloride	0.1
Baking soda	0.2
Premix (micro minerals + vitamins)	0.05
Phytase	0.01
**Total**	**100.0**

Table S4. Nutrient Content of Gestation Feed

Nutrient	Level
Crude protein	15.0%
Crude fat	4.46%
Crude ash	5.76%
Crude fiber	6.16%
Calcium	0.86%
Total phosphorus	0.64%
Sodium	0.24%
Digestible phosphorus	0.35%
Digestible lysine	0.66%
Digestible methionine + cystine	0.50%
Digestible threonine	0.46%
Digestible tryptophan	0.15%
**Net energy**	**2654 kcal/kg**

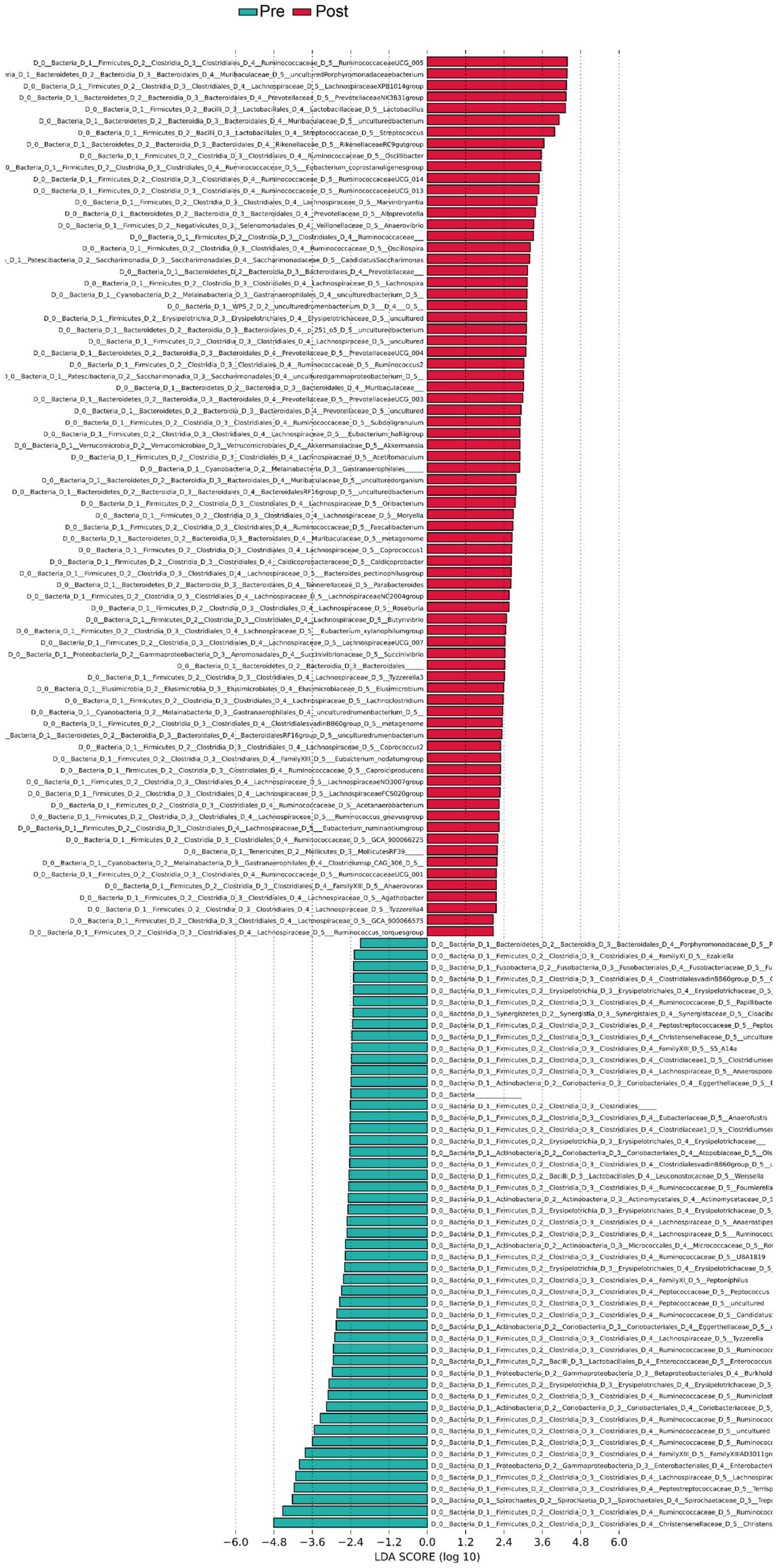

Pre
Post
−6.0 −4.8 −3.6 −2.4 −1.2 0.0 1.2 2.4 3.6 4.8 6.0
LDA SCORE (log 10)

Figure S1. LDA effect size bar plot of differentially abundant microbial taxa between prepartum and postpartum groups (prepartum group, n = 18; postpartum group, n = 18). The figure shows microbial taxa with significant differences between the prepartum and postpartum groups. Red bars represent taxa significantly enriched in the postpartum group, while green bars represent taxa significantly enriched in the prepartum group. The length of each bar corresponds to the absolute value of the LDA score (log10), with larger values indicating stronger effect of the taxa as biomarkers for group differentiation. Differentially abundant taxa were screened using the Kruskal-Wallis test ($P < 0.05$) and LDA analysis (LDA score > 2). Pre: Prepartum group; Post: Postpartum group.
